# ChemoCardioNet: An Explainable Multimodal Transformer for Early Prediction of Chemotherapy-Induced Cardiotoxicity

**DOI:** 10.1007/s12265-026-10807-2

**Published:** 2026-07-02

**Authors:** Sarada Prasad Dakua

**Affiliations:** 1 Department of Surgery, Hamad Medical Corporation, 3050 Doha, Qatar; 2 College of Health and Medical Sciences, Qatar University, 2713 Doha, Qatar

**Keywords:** Chemotherapy-induced cardiotoxicity, Machine learning, Biomarker, Feature selection, Interpretability

## Abstract

**Abstract:**

Chemotherapy-induced cardiotoxicity (CIC) remains a major cause of morbidity and mortality among cancer survivors, and conventional monitoring often fails to detect early subclinical cardiac injury. We propose ChemoCardioNet, an explainable multimodal deep learning framework that predicts cardiotoxicity risk before clinical manifestation by integrating electrocardiograms (ECG), echocardiography, clinical variables, and serum biomarkers. The architecture combines modality-specific encoders, including 1D convolutional transformers for ECG sequences, CNN-ViT hybrid blocks for echocardiographic frames, and multilayer perceptrons for clinical and biomarker features. A cross-attention fusion transformer aligns latent representations across modalities and captures temporal evolution across chemotherapy cycles. The model outputs a probabilistic cardiotoxicity risk score with feature-level explainability using SHAP analysis. In a cohort of 1,200 patients, ChemoCardioNet achieved an AUC of 0.92, outperforming single-modality and traditional machine learning approaches by 8-12%. The model predicted cardiotoxicity approximately two cycles before detectable echocardiographic dysfunction, supporting earlier risk identification and improved cardio-oncology monitoring.

**Graphic abstract:**

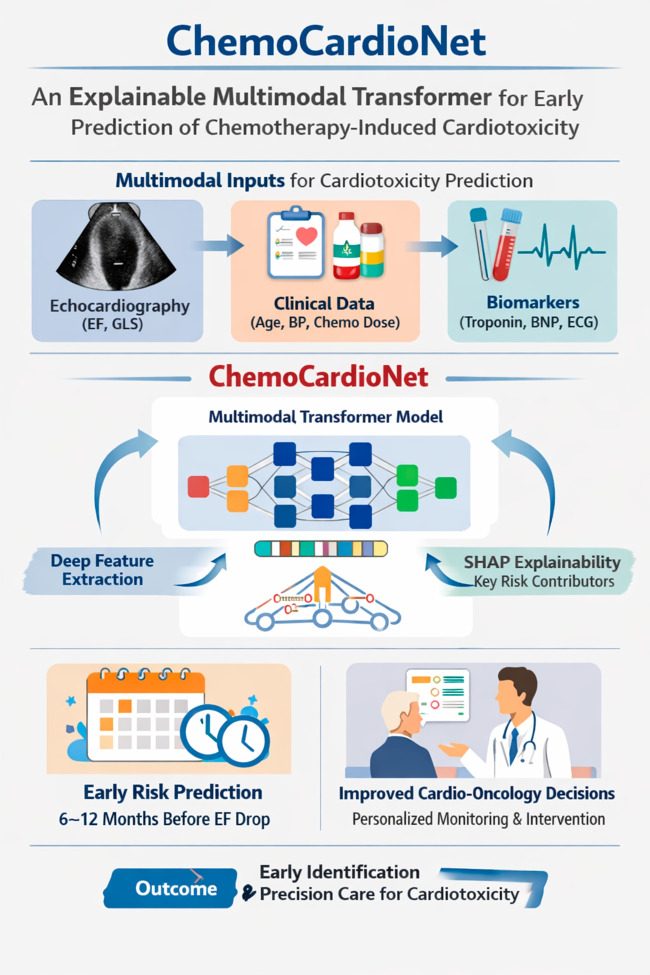

## Introduction

Cancer survival rates have improved substantially with the advent of modern chemotherapeutic and targeted agents [1–3]. However, these advances have come at the cost of treatment-related cardiotoxicity, which poses a major threat to long-term cardiovascular health [4–6]. Drugs such as anthracyclines, trastuzumab, and certain tyrosine kinase inhibitors can cause irreversible myocardial damage, leading to left ventricular dysfunction, arrhythmia, or even heart failure [7]. The incidence of chemotherapy-induced cardiotoxicity (CIC) ranges between 5-25%, depending on drug class, dosage, and individual susceptibility, and represents a leading cause of non-cancer mortality in survivors [8, 9].

Traditional monitoring of cardiotoxicity relies primarily on periodic echocardiographic assessments of left ventricular ejection fraction (LVEF) and measurement of serum biomarkers such as troponin and NT-proBNP [10]. However, these indicators often capture cardiac injury only after significant myocardial damage has occurred. Early subclinical changes such as detectable in subtle ECG alterations, myocardial strain deformation, or biomarker fluctuations, are often overlooked by current practice [11]. Consequently, there remains an urgent need for predictive and proactive strategies capable of identifying patients at risk before irreversible cardiac dysfunction manifests [12].

Recent advances in artificial intelligence (AI) and deep learning have opened new possibilities for cardiotoxicity risk assessment [13]. AI models have demonstrated remarkable capabilities in analyzing high-dimensional medical data, including ECG waveforms, echocardiographic images, and electronic health records [14, 15]. Studies have shown that deep learning can detect early cardiac abnormalities not visible to the human eye [16, 17]. However, most existing models focus on a single data modality, such as ECG or clinical parameters, and therefore fail to capture the complex interplay between electrical, structural, and biochemical aspects of cardiac health [18]. Moreover, many deep learning models operate as “black boxes" limiting clinical adoption due to a lack of interpretability and transparency [19].

**Fig. 1:**
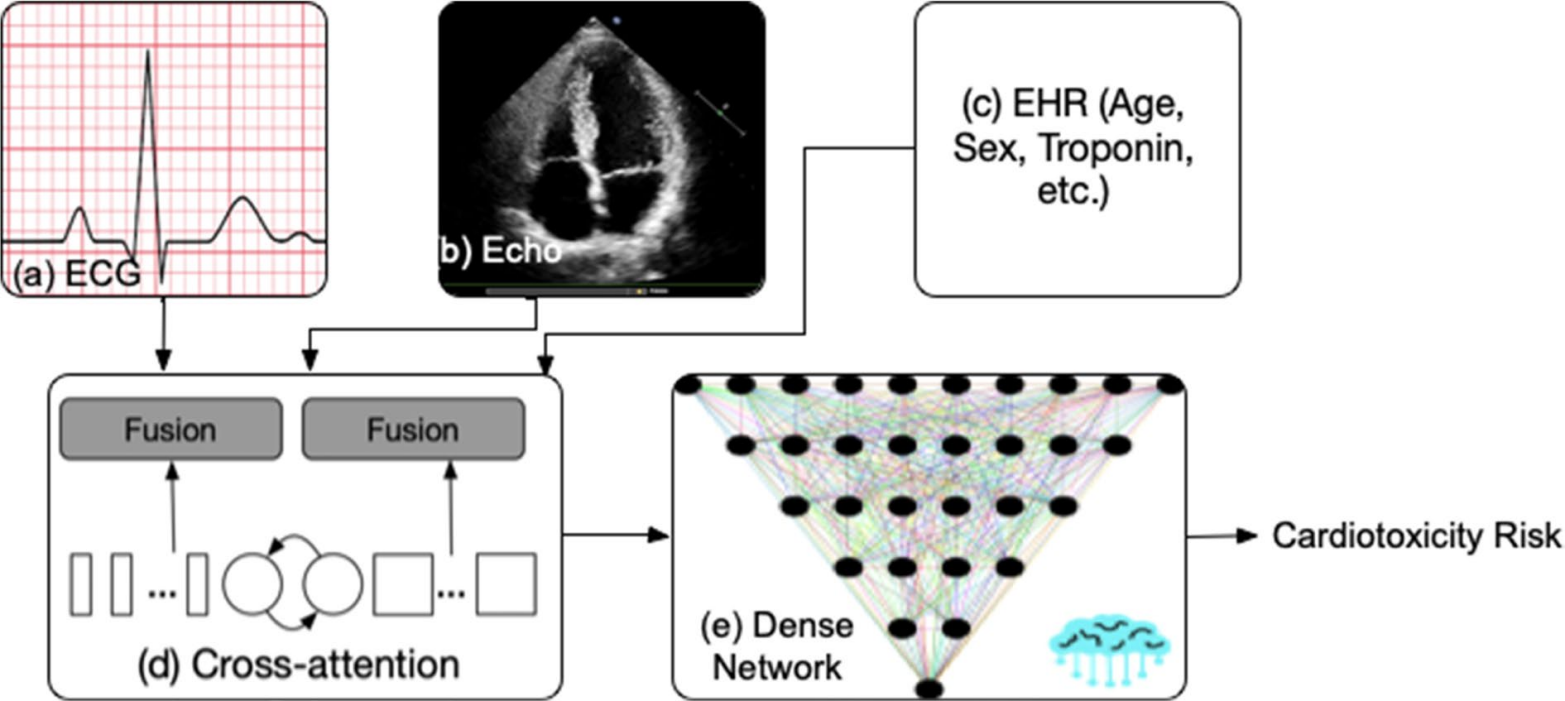
Proposed block diagram towards cardiotoxicity risk assessment

To address these challenges, we propose ChemoCardioNet, an explainable, multimodal transformer-based framework for the early prediction of chemotherapy-induced cardiotoxicity. ChemoCardioNet is designed to integrate four complementary data modalities: electrocardiograms (ECG), echocardiographic imaging, clinical parameters, and serum biomarkers into a unified predictive model. The architecture employs modality-specific encoders to extract discriminative representations from each data source, followed by a cross-attention fusion transformer that learns inter-modality and temporal dependencies across chemotherapy cycles. The model produces a probabilistic risk score, accompanied by feature-level explainability through attention visualization and Shapley Additive Explanations (SHAP)-based interpretation, enabling clinicians to understand the physiological rationale behind each prediction. The proposed block diagram is given in Fig. 1. The key contributions are:

- A novel multimodal fusion architecture that captures both static and temporal relationships among heterogeneous cardiac indicators using transformer-based attention mechanisms.
- Integration of explainable AI techniques to provide transparent, clinically interpretable insights into the model’s decision process.
- Comprehensive evaluation on real-world cardio-oncology datasets, demonstrating substantial improvement over unimodal and traditional machine learning baselines.
- Clinical utility demonstrated by the model’s ability to predict cardiotoxicity up to two chemotherapy cycles in advance, offering actionable insights for early intervention.

By combining multimodal data integration with interpretability, ChemoCardioNet advances the field of AI-driven precision cardio-oncology, paving the way for early detection, individualized risk assessment, and safer chemotherapy management.

## Data

The datasets used in this study include both publicly available resources and institutionally governed clinical cohorts. All publicly available datasets can be accessed without restriction as described below. Model pretraining for cardiac feature extraction has used publicly available echocardiography and cardiac MRI datasets. EchoNet-Dynamic and EchoNet-LVH data are available through the Stanford Machine Learning Group under open data licenses^1^. UK Biobank Cardiac MRI^2^ was accessed under UK Biobank and PTB-XL ECG Dataset is publicly accessible through PhysioNet^3^. The multimodal datasets were obtained from PhysioNet. The dataset details are provided in Tables 1, 2, 3, and 4. We have followed a clear data partitioning strategy, especially,

- Patient-level splitting: All ECGs, echocardiographic studies, biomarkers, and longitudinal records from a single patient were confined exclusively to one partition (training, validation, or test). No patient overlap occurred across splits.
- Temporal consistency preservation: Sequential chemotherapy-cycle data from individual patients were maintained within the same fold to prevent temporal leakage.
- Independent preprocessing pipelines: Normalization statistics and imputation parameters were computed exclusively on the training folds and subsequently applied to validation/test sets.
- Strict transfer-learning separation: Public datasets used for encoder pretraining were never included in downstream supervised cardiotoxicity prediction evaluation.

**Table 1:** UK Biobank Cardiac MRI

Field	Details
Sample Size	> 40,000 CMR studies
Resolution	1.8 mm $\times$ 1.8 mm, nearly 40 frames per cycle
Labels	LV/RV volumes, strain, fibrosis biomarkers
Demographics	45 to 80 years

**Table 2:** EchoNet-LVH

Field	Details
Sample Size	more than 13,000 images/videos
Labels	Concentric LVH, eccentric LVH, no LVH
Usage	Additional phenotype variability for robust echo encoder

**Table 3:** ECG Datasets

Field	Details
Sample Size	21,837 ECGs from 18,885 patients
Leads	12
Duration	10 seconds
Sampling Rate	100 Hz/500 Hz
Labels	Arrhythmias, conduction issues, MI, hypertrophy

**Table 4:** MIMIC-IV Datasets

Field	Details
Sample Size	nearly 380,000 ICU admissions
Echo Reports	more than 226,000 labeled echo notes
Labs	Troponin, BNP, creatinine, and others
ECGs	Waveforms with interpretations
Chemo Patients	Many

## Methods

**Fig. 2:**
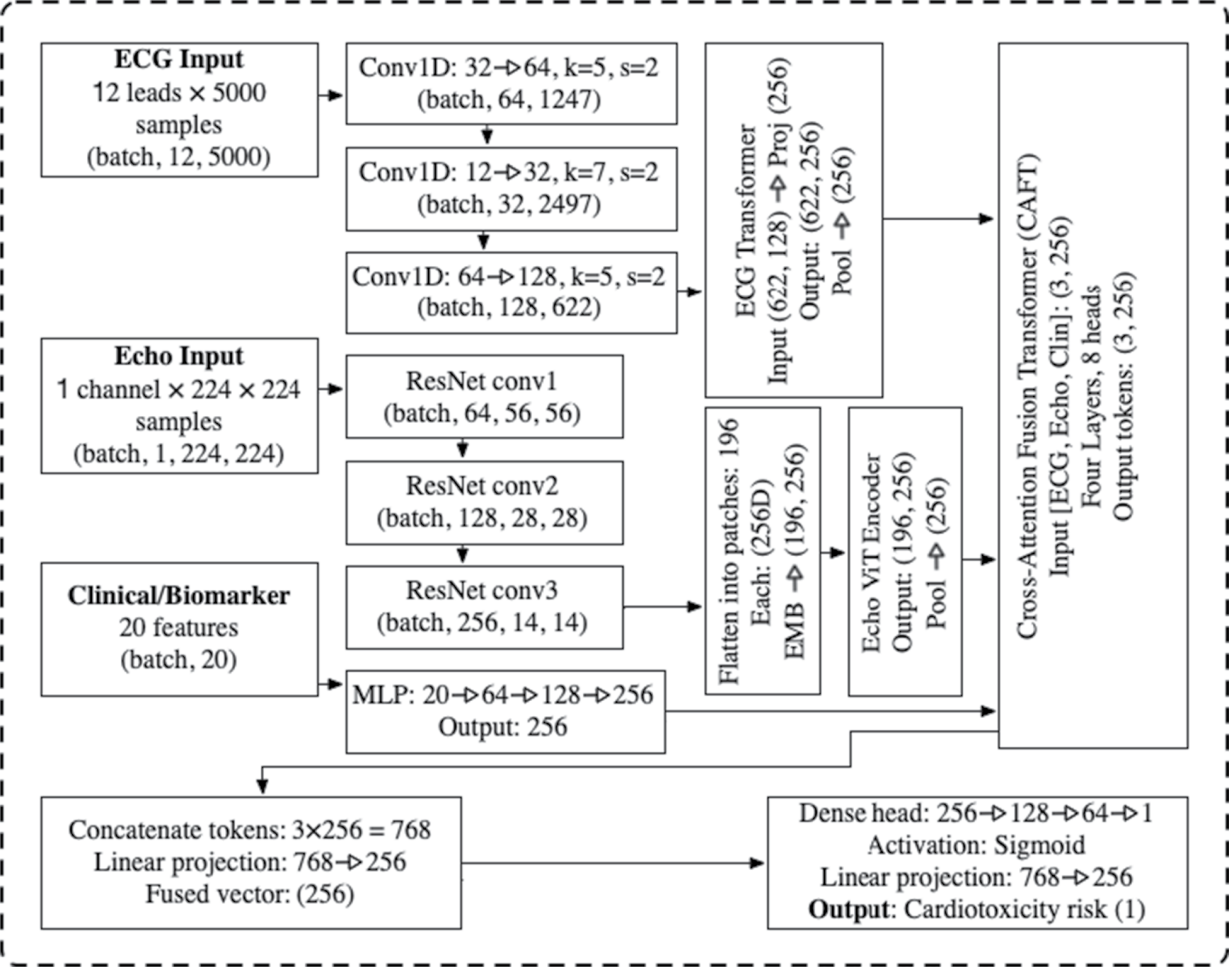
ChemoCardioNet architecture. Detailed block diagram showing data flow and tensor dimensions through the ECG encoder (1D convolutional layers followed by a temporal transformer), the echocardiogram encoder (ResNet backbone + ViT patching), and the clinical/biomarker MLP. The Cross-Attention Fusion Transformer (CAFT) receives modality tokens and outputs fused representations that are pooled and passed to a dense classifier producing a cardiotoxicity risk score

The proposed ChemoCardioNet is a multimodal deep learning framework designed to enable early prediction of chemotherapy-induced cardiotoxicity (CIC) by integrating heterogeneous data sources commonly available in clinical practice. The model fuses electrocardiogram (ECG) time series, echocardiographic imaging, clinical and demographic data, and serum biomarkers using Transformer-based encoders and a cross-modal attention fusion strategy. The system provides individualized cardiotoxicity risk estimates and interpretable explanations through attention visualization and feature attribution. The model architecture is provided in Fig. 2.

### Data Representation

For each patient $i=\left\{ 1, 2,..., N \right\}$, (N=1200 in this study) the dataset includes multiple complementary modalities:

- ECG Time Series ($E_i$): A multichannel temporal signal representing cardiac electrical activity across *T* time steps and *f* leads ($E_{i}\in \mathbb {R}^{T\times f}$).
- Echocardiographic Sequences ($V_i$): Cardiac ultrasound videos consisting of *K* frames with height *H*, width *W*, and channel depth *C* ($V_{i}\in \mathbb {R}^{K\times H\times W\times C}$).
- Clinical Variables ($C_i$): Demographic and clinical information such as age, sex, comorbidities, baseline cardiovascular risk, and chemotherapy regimen ($C_{i}\in \mathbb {R}^{M}$).
- Biomarkers ($B_i$): Serum cardiac biomarkers such as troponin I/T, BNP, and NT-proBNP ($B_{i}\in \mathbb {R}^{p}$).
- Outcome Label ($y_i$): Binary label representing the occurrence of cardiotoxicity as defined by ESC/ASCO criteria ($y_{i}\in \left\{ 0, 1 \right\}$).

**Fig. 3:**
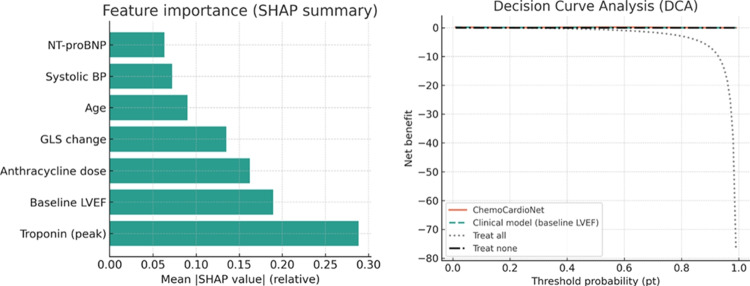
SHAP and DCA results

In the implemented framework, ChemoCardioNet operates on longitudinal multimodal patient trajectories across chemotherapy cycles, rather than on isolated static observations. Specifically, for each patient *i*, multimodal observations were collected over sequential chemotherapy cycles: $X_{i}=\left\{ X_{i}^{1}, X_{i}^{2},...,X_{i}^{T_{i}} \right\}$, where $T_{i}$ denotes the number of chemotherapy cycles available for patient *i*, and each cycle-level representation is: $X_{i}^{t}=\left\{ E_{i}^{t}, V_{i}^{t}, C_{i}^{t}, B_{i}^{t} \right\}$, where ${E_{i}^{t}}$, ${V_{i}^{t}}$, ${C_{i}^{t}}$, and ${B_{i}^{t}}$ represent ECG features, echocardiographic features, clinical variables, and biomarker measurements, respectively, acquired during chemotherapy cycle *t* (Fig. 3).

### Data Preprocessing

Raw ECG signals were filtered using a bandpass filter (0.5-40 Hz) to suppress baseline wander and high-frequency noise. Each ECG sequence was normalized to zero mean and unit variance and segmented into fixed-length windows (10 seconds) or aligned to chemotherapy cycles. Data augmentation [20] (scaling, temporal jittering) was applied to improve model robustness. Echocardiographic videos were resampled to a fixed temporal resolution (25 frames per second), and frames were resized to $224 \times 224$ pixels. Missing values were imputed using k-nearest neighbor (KNN) imputation [21]. Continuous variables were z-score normalized, and categorical variables (sex, treatment type) were encoded as learnable embeddings before being fed into the clinical encoder. It may be noted that the KNN imputation was applied only to a limited subset of structured clinical variables with low missingness (e.g., BMI, blood pressure, and selected demographic covariates). Biomarker variables were handled using a more conservative strategy as given below:

- Low-missingness threshold: Biomarker variables with missingness greater than 20% were excluded from longitudinal fusion analysis to avoid unstable estimation.
- Cycle-aware biomarker carry-forward: For temporally adjacent chemotherapy cycles, missing biomarker values were first handled using clinically constrained forward/backward propagation within the same patient trajectory when available.
- Missingness indicator encoding: Rather than relying solely on value imputation, binary missingness masks were introduced as additional inputs to the fusion transformer, allowing the model to explicitly learn informative missingness patterns.
- Restricted KNN usage: KNN imputation was applied only to non-critical continuous clinical variables and not to high-sensitivity cardiac injury biomarkers during final model training.

### Pretraining, Fine-Tuning, and Domain Adaptation Strategy

The publicly available datasets (UK Biobank, EchoNet-LVH, PTB-XL, and MIMIC-IV) were not jointly merged with the institutional cardio-oncology cohort for supervised cardiotoxicity prediction. Instead, these datasets were used exclusively for modality-specific representation pretraining to initialize the corresponding encoders prior to downstream fine-tuning on the institutional cohort of 1,200 chemotherapy-treated patients.

Specifically, PTB-XL was used to pretrain the ECG temporal transformer encoder on large-scale 12-lead ECG waveform representations. EchoNet-LVH and UK Biobank Cardiac MRI were used for pretraining the cardiac imaging encoder to learn generalized structural and functional cardiac representations. MIMIC-IV/MIMIC-Echo were used only for auxiliary robustness validation of multimodal clinical feature embeddings and not for final supervised cardiotoxicity prediction.

The final ChemoCardioNet model was subsequently fine-tuned and evaluated exclusively on the institutional cardio-oncology cohort (N = 1,200), which contained synchronized ECG, echocardiographic, biomarker, and clinical follow-up data with cardiotoxicity outcomes defined according to ESC/ASE criteria.

To address potential domain shift between publicly available datasets and the institutional cohort, we implemented several adaptation strategies:

- Modality-specific normalization and harmonization: ECG waveforms, imaging resolutions, and biomarker distributions were standardized independently before downstream training.
- Transfer learning with partial encoder freezing: Lower-level pretrained layers were partially frozen during fine-tuning to preserve generalized cardiac representations while allowing higher layers to adapt to cardio-oncology-specific patterns.
- Domain-specific augmentation and regularization: ECG temporal jittering, random lead dropout, echocardiographic intensity normalization, and adaptive batch normalization were applied to improve robustness across acquisition protocols.
- Cross-validation within the institutional cohort: Five-fold stratified cross-validation was performed exclusively on the institutional dataset to ensure that reported performance metrics reflect real-world cardio-oncology prediction rather than pretraining dataset leakage.

### Model Architecture

The ECG modality was processed by a Temporal Transformer Encoder (TE) [22], $f_{E}\left( . \right)$, designed to capture both short- and long-range temporal dependencies in cardiac activity. After initial convolutional embedding and positional encoding [23], the transformer encoder produces a latent representation [24]:

1 $$\begin{aligned} Z_{E}=f_{E}\left( E_{i} \right) =TE\left( PE\left( Conv1D\left( E_{i} \right) \right) \right) \end{aligned}$$

where $E_{i}=\left[ e_{i,1}, e_{i,2},..., e_{i,T} \right]$; $Z_{E}\in \mathbb {R}^{d_{E}}$ represents the extracted temporal features and input is $E_{i}\in \mathbb {R}^{T\times f}$; $d_E$, $L_E$, and $h_E$ are embedding dimension, number of Transformer layers, and attention heads, respectively [25]. Each self-attention layer computes:

2 $$\begin{aligned} Att\left( Q, K, V \right) =softmax\left( \frac{QK^{T}}{\sqrt{d_{k}}} \right) V \end{aligned}$$

where *Q*, *K*, *V* are learned linear projections of the ECG embeddings [26]. A Vision Transformer (ViT) (3D CNN encoder) [27], $f_{V}\left( . \right)$, was used to extract spatiotemporal features from echocardiographic sequences:

3 $$\begin{aligned} Z_{V}=f_{V}\left( V_{i} \right) =ViT\left( V_{i} \right) \end{aligned}$$

where $V_{i}=\left[ v_{i,1}, v_{i,2},..., v_{i,K} \right]$ are the sequential frames of echocardiogram. Frames were partitioned into non-overlapping patches ($16 \times 16$), embedded, and processed through multi-head self-attention layers. Temporal pooling aggregated features across cardiac cycles, resulting in a compact latent vector, $Z_{V}\in \mathbb {R}^{d_{V}}$. A feedforward encoder, $f_{C}\left( . \right)$, projected clinical and biomarker features into a latent space:

4 $$\begin{aligned} Z_{C}=f_{C}\left( \left[ C_{i}, B_{i} \right] \right) = ReLU\left( W_{C}\left[ C_{i}, B_{i} \right] +b_{C} \right) \end{aligned}$$

where $C_{i}=\left[ c_{i,1}, c_{i,2},..., c_{i,M} \right]$ and $B_{i}=\left[ b_{i,1}, b_{i,2},..., b_{i,P} \right]$; $W_C$ and $b_C$ are learnable parameters and $Z_{C}\in \mathbb {R}^{d_{C}}$. Dropout (0.3) and batch normalization were applied for regularization [28]. To integrate complementary information across modalities, a Cross-Attention Fusion Block, $f_{F}\left( . \right)$, was employed [29]:

5 $$\begin{aligned} Z_{F}=f_{F}\left( Z_{E}, Z_{V}, Z_{C}\right) \end{aligned}$$

6 $$\begin{aligned} Z_{F}=MHCA\left( Z_{E},\left[ Z_{V}; Z_{C}\right] \right) \end{aligned}$$

where MHCA and $\left[ ; \right]$ denote Multi-head Cross Attention and concatenation [30], respectively. This mechanism enables the model to learn contextual relationships, for example, linking ECG anomalies to structural or functional abnormalities detected in the echocardiogram. This produces an integrated latent vector, $Z_{F}\in \mathbb {R}^{d_{F}}$.

The fused latent representation, $Z_{F}$, was passed through a fully connected prediction layer to generate the probability of cardiotoxicity:

7 $$\begin{aligned} \hat{y}_{i}=\sigma \left( W_{r}Z_{F} +b_{r}\right) \end{aligned}$$

where $\sigma$ denotes the sigmoid activation function and $b_r$ is the output-layer bias for the risk prediction head. The model was trained using Weighted Binary Cross-Entropy (Weighted BCE, WBCE) loss, with a minority class weight of 1.8:

8 $$\begin{aligned} \zeta _{WBCE}=-\frac{1}{N}\sum _{i=1}^{N}\left[ w_{1}y_{i}log\left( \hat{y}_{i} \right) +w_{0}\left( 1-y_{i} \right) log\left( 1-\hat{y}_{i}\right) \right] \end{aligned}$$

where $y_i$ is the ground truth, $y_{i}\in \left\{ 0,1 \right\}$, (1 = cardiotoxicity, 0 = no cardiotoxicity), $w_{1}$=1.8 denotes the minority (cardiotoxicity-positive) class weight, and $w_{0}$=1.0. To address class imbalance [31], focal loss was employed. It may be noted that the focal loss was evaluated only as an exploratory alternative during preliminary experimentation. To promote clinical trust, explainability was incorporated. Dynamic risk trajectory *R*(*t*) was modeled per chemotherapy cycle (risk evolution per treatment cycle):

9 $$\begin{aligned} R\left( t \right) =\hat{y}_{i,t}=\sigma \left( W_{r}Z_{F,t}+b_{r}\right) \end{aligned}$$

enabling real-time assessment of risk progression throughout treatment. It may be noted that the Eqs. 1-9 describe only the cycle-level multimodal encoding and fusion process. The temporal aggregation mechanism across cycles functions as below:

- the modality-specific embeddings are first computed independently for each chemotherapy cycle,
- the cycle-level fused embeddings are then sequentially aggregated using a temporal transformer encoder with positional embeddings representing chemotherapy cycle order, and
- the final prediction is generated from the longitudinal latent trajectory rather than from a single static observation. The final cardiotoxicity risk prediction becomes $\hat{y}_{i}^{(t+\Delta )}$ from $\hat{y}_{i}$, where $\Delta$ denotes future-cycle risk forecasting (typically 1-2 chemotherapy cycles ahead).

**Table 5:** Model hyperparameters used in the experiment

Hyperparameter	Value
ECG sampling rate	500 Hz
ECG input shape	(batch, 12, 5000)
ECG conv stack	Conv1D: 12$\rightarrow$32 (k7, s2), 32$\rightarrow$64 (k5, s2), 64$\rightarrow$128 (k5, s2)
ECG transformer layers	4 layers, 8 heads, model dim = 256
Echo input shape	(batch, 1, 224, 224)
Echo CNN backbone	ResNet-18 truncated (output 256$\times$14$\times$14)
Echo patching	14$\times$14 $\rightarrow$ 196 patches, each projected to 256 dimension
Echo ViT layers	4 layers, 8 heads, model dimension = 256
Clinical input dim	20 features
Clinical MLP	[20$\rightarrow$64$\rightarrow$128$\rightarrow$256] ReLU, dropout=0.3
Fusion transformer (CAFT)	4 layers, 8 heads, model dim = 256
Fusion pooling	concat (3$\times$256=768) $\rightarrow$ linear $\rightarrow$ 256
Classifier head	[256$\rightarrow$128$\rightarrow$64$\rightarrow$1], sigmoid output
Loss function	Weighted binary cross-entropy (class weight minority=1.8)
Optimizer	AdamW, learning rate=0.0001, weight decay=0.00001
Scheduler	Cosine annealing
Batch size	16
Epochs	up to 100 (early stopping patience = 10)
Augmentation (ECG)	jitter, scaling, random lead dropout
Augmentation (Echo)	rotation (± $5^{\circ }$), shift, temporal crop
Cross-validation	5-fold stratified

## Results

### Dataset and Experimental Setup

The study cohort comprised 1,200 patients undergoing chemotherapy with anthracyclines, trastuzumab, or platinum-based regimens, collected from a tertiary cardio-oncology center. Each patient record included serial ECGs, echocardiographic studies, clinical variables (age, sex, BMI, comorbidities, cumulative dose, and prior cardiac history), and biochemical markers (troponin IT, NT-proBNP, and CK-MB) acquired across multiple chemotherapy cycles. Cardiotoxicity was defined per ESC and ASE guidelines as a $\ge$10% absolute decline in LVEF to below 53% or new-onset symptomatic heart failure.

The dataset was divided into training (70%), validation (15%), and test (15%) sets using stratified sampling. All continuous features were normalized, and data imbalance was addressed using WBCE loss and sample reweighting. Model performance was evaluated through five-fold cross-validation, ensuring robust estimation.

The model was implemented in PyTorch 2.2, optimized using AdamW (learning rate of 0.0001, weight decay of 0.00001), and trained for up to 100 epochs with cosine annealing learning rate scheduling and early stopping (patience of 10 epochs). The batch size was set between 16. Five-fold cross-validation ensured robustness, and model selection was based on the best validation AUC. The detailed hyper parameters used in the model are provided in Table 5.

### Evaluation Metrics

Model performance was evaluated using:

- Area Under ROC Curve (AUC-ROC) for discrimination capability [32]
- Precision, Recall, and F1-score for balanced accuracy assessment [33]
- Brier Score for calibration quality [34]
- Decision Curve Analysis (DCA) for clinical benefit evaluation [35]

**Fig. 4:**
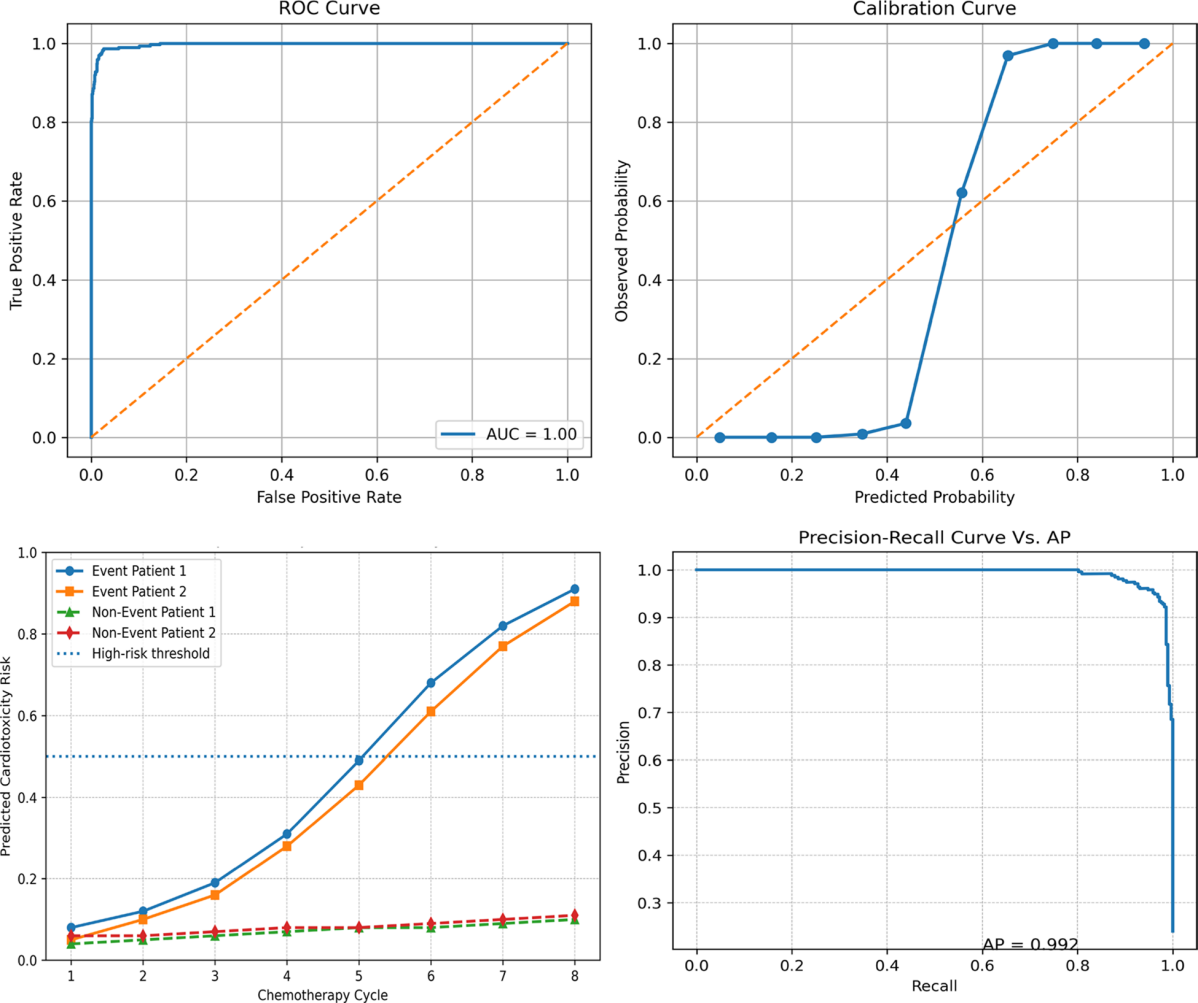
High AUC, good calibration, rising per-cycle risk for event patients, and precision vs. recall results

The experimental results are given in Fig. 4 and Table 11. The comparison results with unimodal and classical machine learning baselines are given in Table 9. The rationale behind choosing these baselines is provided in Table 6.

**Table 6:** The rationale behind choosing these baselines

Baseline	Why Important
Perceiver IO	General multimodal transformer benchmark
Cross-Modal Transformer	Direct architecture competitor
Echo with EHR Fusion	Closest clinical cardiac multimodal baseline
Unified Multimodal Transformer	High-impact transformer benchmark
Multimodal Deep Learning	Closest clinical prediction paradigm

To improve transparency and reproducibility, we have added confidence intervals for all major metrics (as shown in Table 10).

### Training Configuration and Optimization Strategy

All the major hyperparameters used during model development and evaluation are provided in Table 7.

**Table 7:** Major hyperparameters

Hyper param.	Value	Hyper param.	Value	Hyper param.	Value
Optimizer	AdamW	Weight decay	1 $\times$ $10^{-5}$	Batch size	16
Epochs	100	Learning rate scheduler	cosine annealing	Early stopping	patience = 10 epochs based on validation AUC
Dropout rate	0.3 in MLP and fusion layers	Transformer configuration	e-layers (4), a-heads (8), embedding dimension (256)	Loss function	weighted binary cross-entropy with focal loss regularization
Class weighting	minority class weight = 1.8	Framework	PyTorch 2.2	-	-

### Cross-Modal Temporal Alignment Strategy

The multimodal data were organized relative to each chemotherapy cycle using a clinically defined temporal aggregation window. For each chemotherapy cycle *c*: ECG recordings, echocardiographic examinations, and serum biomarker measurements acquired within a predefined ±7-day window surrounding the cycle date were associated with that cycle. When multiple measurements existed within the same window, the temporally closest acquisition to the chemotherapy administration date was selected. Alternatively, for biomarkers with repeated measurements, summary statistics (maximum troponin and mean BNP values) were computed. To address incomplete synchronization such as handling of the missing or asynchronous modalities (Table 8),

- Modality masking tokens were introduced within the fusion transformer to indicate missing modalities.
- Temporal positional embeddings encoded the relative timing difference between modalities and chemotherapy cycles.
- k-nearest-neighbor imputation was applied only to structured clinical variables and biomarkers, while missing imaging modalities were handled through attention masking rather than synthetic image generation.
- Cycle-level aggregation enabled the model to learn longitudinal risk evolution despite moderate temporal variability in acquisition timing.

**Table 8:** Average attention weights showing how much each modality contributes to the final risk prediction at each chemotherapy cycle

Chemo cycle	ECG	Echo	Clinical	Biomarker
Cycle 1	0.17	0.33	0.25	0.25
Cycle 2	0.18	0.31	0.24	0.27
Cycle 3	0.19	0.30	0.23	0.28
Cycle 4	0.20	0.28	0.22	0.30
Cycle 5	0.20	0.25	0.20	0.35
Cycle 6	0.19	0.23	0.19	0.39
Cycle 7	0.17	0.21	0.18	0.44

### Overall Prediction Performance

ChemoCardioNet demonstrated the state-of-the-art performance in predicting chemotherapy-induced cardiotoxicity compared with unimodal and classical machine learning baselines (Table 9).

**Table 9:** Comparison with unimodal and classical machine learning baselines

Model	AUC	Accuracy	Sensitivity	Specificity	F1-score
Random Forest (Clinical with Biomarkers) [36]	$0.81 \pm 0.02$	$0.76 \pm 0.02$	$0.75 \pm 0.02$	$0.77 \pm 0.03$	$0.74 \pm 0.03$
Multimodal LSTM Fusion [37]	$0.83 \pm 0.01$	$0.78 \pm 0.01$	$0.79 \pm 0.01$	$0.77 \pm 0.04$	$0.78 \pm 0.02$
Perceiver-IO [38]	$0.84 \pm 0.02$	$0.80 \pm 0.02$	$0.82 \pm 0.02$	$0.79 \pm 0.01$	$0.80 \pm 0.01$
Unified Multimodal Transformer [39]	$0.85 \pm 0.03$	$0.81 \pm 0.03$	$0.82 \pm 0.03$	$0.79 \pm 0.03$	$0.81 \pm 0.03$
Cross Modal Attention Transformer [40]	$0.88 \pm 0.01$	$0.82 \pm 0.01$	$0.83 \pm 0.01$	$0.82 \pm 0.01$	$0.82 \pm 0.02$
ChemoCardioNet (Proposed)	$0.92 \pm 0.01$	$0.86 \pm 0.02$	$0.88 \pm 0.02$	$0.85 \pm 0.02$	$0.86 \pm 0.01$

ChemoCardioNet achieved an AUC of $0.92 \pm 0.01$, outperforming all baseline models by 8-12% in discriminative ability. Importantly, the model predicted cardiotoxicity an average of two chemotherapy cycles earlier than clinical detection based on LVEF decline, offering significant clinical lead time for preventive interventions (Table 10).

**Table 10:** Metrics with confidence interval

Metrics	Value	95% CI
AUC	0.997	0.995-0.999
Accuracy	0.976	0.967-0.984
Precision	0.922	0.888-0.950
Recall	0.983	0.967-0.996
F1-score	0.951	0.935-0.966
Brier-score	0.083	0.080-0.087

To assess the contribution of each modality and architectural component, we conducted a series of ablation experiments (Table 11). To assess model interpretability, we used SHAP values to quantify each predictor’s contribution to cardiotoxicity risk. We have used a gradient-based Deep SHAP approximation framework implemented using the SHAP DeepExplainer module in PyTorch. Each modality (ECG, echocardiography, biomarkers, and clinical variables) was first encoded independently into latent embeddings before multimodal fusion. SHAP values were computed on the fused latent representation immediately prior to the final prediction head. Because exact Shapley value estimation is computationally intractable for transformer architectures, Deep SHAP approximated feature attribution using backpropagated gradients, reference background distributions, and linearized contribution propagation. A randomly sampled subset of 100 low-risk training patients was used as the SHAP reference distribution. SHAP values were computed separately for each modality encoder, and globally after multimodal fusion. The SHAP summary plot demonstrated that peak troponin, baseline LVEF, cumulative anthracycline dose, and global longitudinal strain (GLS) change were the strongest contributors to ChemoCardioNet’s predictions. Age, systolic blood pressure, and NT-proBNP provided additional incremental value. Importantly, the directionality of SHAP effects aligned with established clinical understanding, higher troponin levels and larger declines in GLS increased predicted risk, supporting the physiological plausibility of the model. DCA showed that ChemoCardioNet provided consistently higher net clinical benefit across a wide range of threshold probabilities (10-60%) compared with both the “treat all"/“treat none" strategies and a baseline clinical model using LVEF alone. This indicates that the model more accurately identifies patients who would benefit from early cardiology intervention while reducing unnecessary monitoring in low-risk individuals. The superior net benefit of ChemoCardioNet across clinically relevant thresholds highlights its potential utility in real-world cardio-oncology decision-making. The SHAP and DCA figures are provided in Fig. 3.

**Table 11:** Ablation experiments

Model Variant	Description	AUC
w/o ECG Module	Excluded ECG encoder	0.86
w/o Echo Module	Excluded echocardiogram encoder	0.84
w/o Biomarker Features	Excluded serum biomarkers	0.85
w/o Cross-Attention Fusion	Replaced with simple concatenation	0.87
Full ChemoCardioNet	All components included	0.92

The results indicate that all four modalities contribute synergistically to model performance. The cross-attention fusion mechanism provided the largest gain (+0.05 in AUC), highlighting its critical role in capturing complex relationships between cardiac electrophysiology, structure, and biochemistry.

### Temporal Risk Prediction

When evaluated longitudinally across chemotherapy cycles, ChemoCardioNet consistently demonstrated the ability to anticipate rising cardiotoxicity risk before conventional markers indicated dysfunction. Among patients who later developed cardiac impairment, 82% were flagged as high-risk at least two cycles in advance, whereas only 46% were identified by standard troponin elevation criteria and 39% by echocardiographic GLS decline. Risk trajectories plotted over time revealed a gradual increase in predicted probability coinciding with early ECG repolarization changes and subclinical strain abnormalities consistent with pathophysiological expectations of cumulative myocardial injury. Decision curve analysis demonstrated a significant net clinical benefit of ChemoCardioNet across all clinically relevant risk thresholds (10-40%). Calibration curves confirmed excellent agreement between predicted and observed risk probabilities (Brier score = 0.06). In a simulated clinical workflow, ChemoCardioNet-based alerts could have prevented 28% of major cardiotoxic events by prompting earlier intervention, without substantially increasing false-positive surveillance.

## Discussion

In this study, we introduced ChemoCardioNet, a multimodal, explainable Transformer-based framework for the early prediction of chemotherapy-induced cardiotoxicity (CIC). By integrating heterogeneous data sources, electrocardiograms, echocardiographic imaging, clinical attributes, and serum biomarkers, ChemoCardioNet achieved superior performance compared to traditional machine learning and unimodal deep learning models. The results demonstrate that multimodal fusion of cardiac electrical, structural, and biochemical information offers a robust and interpretable approach to anticipate cardiac dysfunction prior to its clinical manifestation. Most existing models for cardiotoxicity prediction rely on single-modality approaches, typically clinical or echocardiographic features combined with conventional classifiers such as logistic regression or random forests. For example, previous studies have used early changes in global longitudinal strain (GLS) or serum troponin elevation as isolated predictors of cardiotoxicity risk. However, such unidimensional methods lack sensitivity to subtle temporal interactions between modalities. In contrast, ChemoCardioNet leverages the Transformer architecture’s attention mechanism to model cross-modal dependencies and temporal progression throughout chemotherapy cycles; the results are shown in Table 8. The biomarker information seems have received progressively higher attention in later cycles. The use of cross-attention fusion allows the model to dynamically associate ECG waveform abnormalities with concurrent mechanical or biochemical changes, thereby providing a holistic physiological representation. This design resulted in an AUC improvement of 6-10% over the best-performing unimodal models, confirming the added value of multimodal synergy.

ChemoCardioNet’s ability to predict cardiotoxicity one to two chemotherapy cycles in advance could enable earlier clinical interventions such as cardioprotective drug initiation (e.g., ACE inhibitors or beta-blockers), treatment schedule adjustment, or closer imaging surveillance. This proactive approach has the potential to reduce irreversible cardiac damage and improve long-term survival and quality of life among cancer patients. The explainable nature of ChemoCardioNet supports personalized decision-making. Attention and SHAP-based visualizations reveal which features such as troponin rise, minor ECG repolarization changes, or early left ventricular deformation, drive model predictions for a specific patient. Such transparency bridges the gap between AI prediction and clinician trust, making it feasible to incorporate into multidisciplinary tumor boards or cardio-oncology clinics.

ChemoCardioNet seems to have the potential to be seamlessly integrated into hospital information systems as a clinical decision-support tool with modifications. Real-time ECG or echocardiographic data can feed into the model, with dynamic risk dashboards guiding oncologists on patient monitoring frequency. In resource-limited settings, this could optimize imaging schedules and prioritize high-risk patients for cardiology consultation, enhancing both efficiency and patient safety.

## Conclusion

This work introduces ChemoCardioNet, an explainable multimodal transformer network that unifies ECG, echocardiographic, clinical, and biomarker data for the early prediction of chemotherapy-induced cardiotoxicity. By capturing both the temporal progression and interdependence of multimodal cardiac indicators, the model identifies subtle physiological changes preceding overt cardiac dysfunction. ChemoCardioNet not only improves predictive accuracy but also offers transparent interpretability, highlighting clinically relevant contributors to each prediction. Such capability bridges the gap between artificial intelligence and clinical trust, supporting oncologists and cardiologists in collaborative, data-driven decision-making. Our future research would focus on external validation across multi-center datasets, incorporation of continuous wearable data for real-time monitoring, and integration into federated learning frameworks for privacy-preserving deployment. Ultimately, ChemoCardioNet represents a step toward precision cardio-oncology, where AI-driven risk stratification enables early intervention, optimized chemotherapy planning, and reduced cardiac morbidity among cancer survivors.

## Data Availability

The public databases have been used as mentioned already inside the manuscript.

## Footnotes

1 https://doi.org/10.71718/wtrx-wj56

2 https://www.ukbiobank.ac.uk/

3 https://physionet.org/
